# 

**Published:** 1887-07

**Authors:** 


VOL. XVII. JULY, 1887. NO. 7.

LIBRARY INDEXED S.G.O.

### THE SOUTHERN MEDICAL RECORD.

EDITORS:

T. S. POWELL, M. D. R. C. WORD, M. D.

COLLABORATORS:

J. McF. GASTON, M. D.,
W. D. BIZZELL, M. D.,
W. P. NICOLSON, M. D.,
E. VAN GOIDTSNOVEN, M. D.,
V. O. HARDON, M. D.,
G. G. ROY, M. D.,
A. G. HOBBS, M. D.,
W. S. ELKIN, M. D.,
W. A. CROW, M. D.,
JNO. THAD. JOHNSON, M. D.

Address R. C. WORD, M.D., Managing Editor, Atlanta, Ga.

Published and Entered as Second-class Matter at ATLANTA, GA.

